# Twelve-Weeks of Bench-Step Exercise Training Ameliorates Cardiopulmonary Fitness and Mood State in Patients with Schizophrenia: A Pilot Study

**DOI:** 10.3390/medicina57020149

**Published:** 2021-02-07

**Authors:** Yu-Chi Kuo, Dan-Yan Chang, Yi-Hung Liao

**Affiliations:** Department of Exercise and Health Science, National Taipei University of Nursing and Health Sciences, Taipei 11219, Taiwan; yuchi.kuo@gmail.com (Y.-C.K.); helloimden@gmail.com (D.-Y.C.)

**Keywords:** 6-min walk distance (6MWD), beck depression inventory-II (BDI-II), symbol digit modalities test (SDMT), mood states, fitness, attention

## Abstract

*Background and objectives:* Unhealthy, physically inactive lifestyles increase the risk of future cardiovascular events and impaired physical fitness in individuals with schizophrenia. Insufficient literature exists to provide fundamental information about appropriate exercise training modality for this population. This pilot study preliminarily investigated the effects of a 12-week moderate-intensity bench-step exercise training (BSET) program on cardiopulmonary fitness, mood state, and cognition in patients with schizophrenia. *Methods:* Twenty-eight patients with schizophrenia completed this study. The participants were allocated into either bench-step exercise-training (BSET; *N* = 14) or control (CTRL; *N* = 14) groups according to their preferences. The BSET group received a 12-week bench-step intervention, whereas the CTRL group did not participate in any training. The Beck Depression Inventory-II (BDI-II), 6-min walk test (6MWD), and Symbol Digit Modalities Test (SDMT) were assessed at baseline (PRE) and at the end of the intervention (POST) to determine mood state, endurance fitness, and attention, respectively. *Results:* After a 12-week BSET intervention, the 6MWD was significantly increased in the BSET (*p* = 0.007) but not in the CTRL (*p* > 0.05). The participants with BSET intervention showed a significant decrease in BDI-II at the end of the intervention (*p* = 0.03). However, SDMT scores were not different in both BSET and CTRL (*p* > 0.05). *Conclusions:* This study demonstrated that the 12-week intervention of moderate-intensity bench-step exercise training (frequency: 1 session/week; each session of 30 min; step cadence: 96 beats/min) might effectively enhance cardiopulmonary fitness and mood state in patients with schizophrenia. However, attention did not change after the bench-step exercise intervention.

## 1. Introduction

Evidence indicates that 1 in 100 individuals may develop a mental disorder at a certain point in their life [1]. Currently, the global prevalence of mental disorders is approximately 1%, with an incidence rate of 0.02–2.16 per 1000 individuals [2]. Of these, approximately 250 million individuals have schizophrenia [3]. Schizophrenia is characterized by thinking and cognitive impairments and behavioral and psychiatric symptoms such as hallucinations (auditory or visual) or delusions (persecutory or reference). As the disease progresses, patients with schizophrenia develop chronic symptoms, including persistent negative symptoms and cognitive impairments, often resulting in a permanent disability that burdens the patients and their family members as well as the healthcare system [3].

Several unhealthy lifestyles, including sedentary behavior or physical inactivity, have commonly been observed in individuals with schizophrenia. Such behavioral features could lead to the rapid development of future cardiovascular events and metabolic disorders [4,5,6,7]. Antipsychotics are commonly used for controlling the related symptoms in individuals with schizophrenia [8]. It has been reported that the use of antipsychotics is associated with the prevalence of metabolic disorders [9,10]. More importantly, there is a higher prevalence of diabetes mellitus [11,12], overweight and metabolic diseases [13], and cardiovascular respiratory diseases [14,15] in the population with schizophrenia.

Cognitive impairments perturb patients with schizophrenia from living independently. Studies have employed neuropsychological testing to verify that these patients have general cognitive impairments [16]. Moreover, impairment in attention/concentration in patients with schizophrenia also results in impeding them from learning complex work skills [17,18]. Patients with schizophrenia may develop obesity-related degenerative disorders (e.g., diabetes, cardiovascular diseases, hypertension, and other chronic diseases) due to unhealthy living behaviors (e.g., smoking, alcohol consumption, and sedentary lifestyle) as well as side effects from antipsychotic agents. These factors all potentially reduce their cardiopulmonary fitness and increase their mortality by two to three times compared with healthy general individuals [19,20,21,22]. In this regard, it would be important for clinical professionals to develop effective non-pharmaceutical practices to better attenuate these negative impacts from schizophrenia.

Exercise training can enhance the physical fitness of patients with schizophrenia. For instance, Scheewe et al. (2013) reported that participants with schizophrenia enrolled in muscle endurance exercise training enhanced their cardiopulmonary endurance and quality of life (QoL). Additionally, the training alleviated their negative symptoms, depression, and anxiety [23]. Acute high-intensity exercise has been reported to show worse cognitive performance compared with moderate-intensity exercise and sedentary controls [24,25]. For relative lower intensity exercise, exercise duration may be extended after patient safety and experience are considered [26]. For example, McDevitt et al. administered 12-week walking exercise training (three sessions weekly, each session of 60 min) to patients with severe mental disorders and found that this intervention enhanced patients’ emotional and social function and that adequate exercise could ameliorate negative symptoms in patients with schizophrenia [27]. The relationship between cognitive function and exercise intensity can be represented as an inverted U-shaped curve [25], suggesting that the use of moderately intense exercise could better enhance cognitive function in patients with schizophrenia.

Taken together, the benefits of exercise on physiological and psychological health have been well recognized. Although exercise in clinical practice is currently available for patients with schizophrenia, studies on relevant routine exercise interventions with fixed intensity and desirable accessibility have been scant. Most of the aforementioned relevant studies were based on relatively low-intensity exercises (e.g., walking exercise training) [23,27]. Consequently, these exercises may be insufficient for fulfilling the treatment need for the moderate-intensity exercise of patients with schizophrenia [26]. More importantly, however, high-intensity aerobic training (85–95% heart rate reserve [HRR]) leads to reduced patient participation rates with schizophrenia (24% attrition rate) [28]. Therefore, we proposed this pilot study to preliminarily investigate the effects of a 12-week moderate-intensity bench-step exercise training (BSET; frequency: 1 session/week; each session of 30 min; step cadence: 96 beats/min) on cardiopulmonary fitness, mood state, and cognition in patients with schizophrenia. We hypothesized that a 12-week intervention of bench-step exercise can effectively alleviate psychiatric syndromes and enhance aerobic capacity in patients with schizophrenia.

## 2. Materials and Methods

### 2.1. Participants and Ethical Statement

This study recruited thirty patients diagnosed as having chronic schizophrenia from a community halfway house. Subsequently, we grouped the participants into bench-step exercise-training (BSET) and control (CTRL) groups according to their preference. The reason to allocate the participants was based on their preference due to the clinical condition with chronic schizophrenia, thus we were not to be able to perform the randomized allocation in this study. After the recruitment, there were 30 eligible participants enrolled in this study, and each group was comprised of 15 participants. During the intervention, there was one participant from each group that dropped out of the study due to personal reasons. All exercise interventions were supervised by a registered occupational therapist, and only participants who attended all twelve times training (frequency: once a week) were included in the final analysis, thus the exercise training compliance ratio was 100%. For the drop-out ratio, one from each group dropped out due to their personal reason during the intervention, thus the drop-out ratio was 6.67% (2 of 30 participants). In total, 28 participants completed the study. The participants were included only if they: (1) were aged 20–65 years; (2) diagnosed as having schizophrenia according to the Diagnostic and Statistical Manual of Mental Disorder, Fifth Edition, Text Revision (DSM-V-TR; APA, 2013); and (3) had diagnosed schizophrenia for 2 years or more. In brief, the DSM-V-TR diagnosed criteria for schizophrenia include: (i) delusions; (ii) hallucinations; (iii) disorganized speech; (iv) grossly disorganized or catatonic behavior; and (v) negative symptoms, and two or more of the above symptoms (>1 month) and at least one of them must be (i), (ii), or (iii). Moreover, some signs of the above disorder must last for a continuous period of at least 6 months for the DSM-V-TR standards. The participants were excluded if they: (1) were unable to follow verbal commands or perform exercise training (both indicating considerable interference in language, cognitive, behavioral, and mental conditions); and (2) had a history of epilepsy, heart disease, and pulmonary function impairment and untreated hypertension. All participants completed a written informed consent form (ICF) and a self-screening health questionnaire (Physical Activity Readiness Questionnaire, PAR-Q) before enrolling in the study. For the procedure of signing written informed consent, in addition to the researcher explaining the entire study process, the research crew members also asked the potential participants to repeat explaining the process to ensure that the potential participants understood all of the experimental procedures and what they need to cooperate during the intervention. This study was conducted with the approval of the National Taiwan University Research Ethics Committee (NTU-REC No: 201703EM012; date: 9 May 2017).

### 2.2. Experiment Design and Procedure

The BSET group received a 12-week intervention of bench-step exercise training, whereas the CTRL group did not receive any type of exercise-training. One day before the intervention, the participant’s baseline characteristics, including height, weight, blood pressure, and heart rate, and Beck Depression Inventory-II (BDI-II) score and Symbol Digit Modalities Test (SDMT) were acquired in the pretest (PRE). Moreover, for the cardiopulmonary/endurance fitness assessment, we instructed the participants to perform a 6-min walking test (6MWD). At the end of the intervention, the aforementioned values were measured again in a posttest (POST). The POST was performed one day after the last intervention to avoid possible influence from the previous exercise session. All test procedures were identical between pretest (PRE) and posttest (POST).

### 2.3. Bench-Step Exercise Training

Our 12-week bench-step exercise training was adapted from a previous study [29] with minor modifications with adjusting the time of warm-up and cool-down exercise. An entire training session lasted 30 min. The sessions were conducted weekly for 12 weeks. In brief, before each training session started and ended, the participants were asked to perform 5-min warm-up and 5-min cool-down exercises. These exercises included 5 min of stretching exercises, static stretching exercises (each movement lasted for 10 s; stretching parts: head, neck, spine, and upper and lower limb) were used, and the stretching movements included flexion, extension, adduction, abduction, rotation, and circumduction. After completing the warm-up exercises, the participants performed 20-min bench-step exercise training beginning at 96 beats/min on a 10-cm-high step (24 sets of stepping up and down the step), which had been shown to elicit an intensity of 40–60% HRR [29]. For every participant, the exercise intensity was increased when their exercise capacity improved. During each training session, the trainer consistently asked the participants’ subjective rate of perceived exertion (RPE; 0–10 scale), and their perceived effort should be within the RPE range of 3–4 to ensure the target intensity (equivalent to 40–60% HRR) were reached. According to the participants’ real-time perceived effort responses, the exercise intensity was personalized by the trainer’s vocal guidance to change their arm-swinging frequencies to reach the appropriate training pace.

### 2.4. Six-Minute Walk Distance Test (6MWD)

The 6-min walking distance test (6MWD) is commonly used to determine whether a participant can complete daily tasks in their living environments. Previous studies have indicated that the 6MWD provides reliable data on physical performance and the clinical functionality of patients with schizophrenia. The 6MWD is capable of rapidly evaluating the effects of moderate- and high-intensity exercises on these patients [6,7,30,31]. Moreover, the 6MWD is a cost-effective, simple, convenient, and rapid test for evaluating the functional performance of patients with schizophrenia [30]. In clinical practice, the 6MWD is used to evaluate aerobic endurance according to the standard procedures detailed by Stookey et al. [32]. Here, our participants were asked to walk at their highest speed possible on a 100-foot-long flat ground surface for continuous 6 min. During the 6MWD test, a research crew guided the participant in completing the test by providing accurate instructions and positive feedback.

### 2.5. Symbol Digit Modalities Test (SDMT)

Symbol Digit Modalities Test (SDMT) is a written test to assess cognitive functioning [33]. The participants were instructed to pair a series of eight symbols with their corresponding numerical digits using a key at the top of the test page. In brief, the participants were required to fill in the boxes under each symbol with their corresponding numerical digit. The total number of these boxes was 110. The test duration was 90 s. The number of items answered correctly within these 90 s indicated the participant’s processing speed. Because SDMT test provided hints to reduce the amount of participant memory required for answer memorization. Therefore, SDMT mainly assessed the selective attention of the participants in this present investigation.

### 2.6. Beck Depression Inventory-II (BDI-II)

BDI-II is a self-reported depression inventory [34]. The participants were asked to score 21 depression-related items on a 4-point scale (0–3). Those who scored 0–13, 14–19, 20–28, and 29–63 points were determined to have minimal, mild, moderate, and severe depression, respectively. In clinical practice, BDI-II is considered to reflect only the degree of depression and thus is not used as a depression diagnosis instrument. However, the BDI-II manual suggests that this inventory reflects the cognitive and emotional status of patients with depression, as well as their somatic and vegetative status.

### 2.7. Statistical Analysis

The commercially available statistical software SPSS 20.0 (version 20.0; IBM Corp., Armonk, NY, USA) was used for statistical analysis. The normality of all measured variables was analyzed using the Shapiro–Wilk normality test provided by SPSS 20.0 software. The independent sample *t*-test and the chi-square test were used to compare the baseline characteristics between both groups. A two-way analysis of variation (2-way ANOVA) was subsequently employed to compare the SDMT, 6MWD, and BDI-II results between both groups. Post hoc analysis using the Bonferroni correction was adopted to detect interactions if the interaction was significant or the parameters under the same categories were compared. The statistical significance level was set at *α* = 0.05, and all statistical data are presented as means ± standard deviations. The effect size (*η*^2^) are categorized as small: <0.01; medium: 0.06; or large: >0.14. The effect size (Cohen’s d) are categorized as small: 0.2–0.3; medium: ≈0.5; or large: ≥0.8. 

## 3. Results

### 3.1. Basic Characteristics of Participants

All of the population characteristics and distribution are shown in Table 1. The gender distribution was the same for both males and females (50% each). The SDMT scores were mostly distributed at the subgroup range with 21–30 and 31–41 scores. The BDI-II score was distributed within 0–13, and the 6MWD distance ranged within 375–449.5 m (11, 39.3%). The basic characteristics of the participants are displayed in Table 1. The average participant age was 52.1 ± 11.0 years old (BSET) and 50.1 ± 8.3 years old (CTRL). The average participant BMI was 24.7 ± 4.6 kg/m^2^ (BSET) and 24.7 ± 3.8 kg/m^2^ (CTRL). The BDI-II assessment score at participant baseline was 11.3 ± 11.6 (BSET) and 10.9 ± 9.5 (CTRL); moreover, the SDMT attention assessments did not show a difference between both groups (BSET: 30.7 ± 9.8 tasks; CTRL: 33.2 ± 10.7 tasks). The distance of 6MWD was 402.4 ± 78.9 m (BSET) and 418.1 ± 55.3 m (CTRL). There were no significant differences between the BSET group and CTRL group in all baseline characteristics.

### 3.2. MWD Endurance Performance after BSET Training

The walking distance (m) for the 6-min walking distance (6MWD) has been widely used as the cardiorespiratory fitness/endurance index. This also reflects the capacity for conducting daily life activities. The 6MWD results are displayed in Table 2. The walking distance for the 6MWD was measured at baseline (PRE) and after the intervention (POST). There was no interaction between the two groups, but there was a time effect (*power = 0.156; F(1,28) = 0.951*; *η^2^ = 0.033*, *small-to-medium ES*; *p < 0.05*). We further compared the differences between PRE and POST within each group and found that, after 12-week bench-step exercise training intervention, the 6MWD distance performance was significantly increased in the BSET group (*p = 0.007; Cohen’s d effect size = 0.424, medium ES*) but not in the CTRL group (*p > 0.05*).

### 3.3. The BSET Training Effects on Mood State (BDI-II) and Attention (SDMT)

The mood of patients with chronic schizophrenia was tested using the Beck Depression Inventory-II (BDI-II) (Table 2). There was a time effect (*power = 0.487; F(1,28) = 3.985; η^2^ = 0.125, medium-to-large ES; p < 0.05*) but not a treatment effect (*p > 0.05*) in BDI-II. The participants with BSET training intervention showed a decrease in BDI-II from 11.4 ± 12 points at baseline (PRE) to 8.8 ± 9.6 points at the end of the intervention (POST) (*p = 0.03; Cohen’s d effect size = 0.235, small ES*). However, there were no differences in BDI-II after the intervention (PRE: 10.9 ± 9.5 points vs. POST: 10.6 ± 8.7 points) compared to the CTRL group (*p > 0.05*). On the other aspect, after 12 weeks of BSET intervention, the corrected task number of symbolic digital module test (SDMT) scores were not statistically different between the two different test stages in both the BSET group and CTRL group (*p > 0.05*) (Table 2).

## 4. Discussion

We administered a 12-week moderate-intensity bench-step exercise training intervention, including 30-min sessions performed once weekly. The primary findings of this study are that 12-weeks endurance exercise training effectively enhanced their mood state and cardiopulmonary fitness/endurance, measured using 6MWD, in patients with chronic schizophrenia. However, the attention level did not exhibit significant enhancements after the exercise intervention compared with the pretest baseline values.

The main objective of schizophrenia treatment is to improve the functional prognosis [35]. Recent clinical research on schizophrenia has focused on metabolic health enhancement and the alleviation of cardiovascular comorbidities [15,36,37,38]. Patients with schizophrenia have been reported to have an increased rate of premature mortality due to cardiovascular comorbidities [39]. Epidemiological evidence reveals that individuals with schizophrenia exhibited three times the risk of suffering sudden cardiac death than those from the general population [40,41]. Additionally, patients with schizophrenia receiving antipsychotics exhibited a very high incidence of metabolic disorders and increased cardiovascular risk [42]. The correlation between schizophrenia and metabolic and cardiovascular diseases mainly involves complex environmental and health factor interactions (i.e., physical activity, acquired nutrition, and drug use) [19,20,21,22]. Hence, it has been suggested that regularly re-evaluating the cardiovascular risk and initiation of behavioral changes are critical to maintaining the health status in the schizophrenia population [43]. Vancampfort et al. (2011) indicated that the amount of physical activity (e.g., walking distance) during leisure time is positively correlated to the health-related QoL of patients with schizophrenia [7]. Therefore, assessing exercise capability is crucial to understanding the effects of schizophrenia and developing healthcare management methods for patients with schizophrenia [6,7,31,44]. Studies indicated that the 6MWD provides data on physical performance and clinical functions of patients with schizophrenia and thereby serves as an effective, simple, convenient, and rapid test to evaluate the effects of moderate- and high-intensity exercises on these patients [6,7,30,31]. In addition, patients with schizophrenia with regular exercising habits and without any metabolism disorder have long walking distances on the 6MWD [44]. Notably, in this study, we employed the 6MWD to evaluate the aerobic capacity of patients with schizophrenia and found that our 12-week intervention enhanced the patients’ aerobic endurance and fitness effectively. Therefore, weekly 30-min sessions of moderate- to high-intensity exercises can enhance the aerobic endurance and exercise capabilities of patients with schizophrenia. Moreover, several lines of evidence reveal that people with schizophrenia are less active and exercise less than those from the general population [4,6,7]. In this regard, because physical exercise capabilities and health-related QoL have been confirmed to be correlated positively [7], we suggest that patients with schizophrenia should conduct at least 30 min of moderate- to high-intensity exercises every week to alleviate cardiovascular and metabolic disorders and maintain physical health.

Empirical intervention studies have proven and supported the benefits of exercise in patients with mental disorders [45,46]. A comprehensive analytical study revealed that weekly interventions of moderate- to high-intensity exercises for 90 min significantly alleviated psychiatric symptoms [46]. Donaghy reviewed 11 prospective longitudinal studies and revealed that exercise training is an effective intervention strategy to mitigate depression. In addition, exercise is a complementary treatment strategy for mild and moderate depression and can serve as a nonpharmacological treatment method to enhance cognitive functioning [45]. Most studies have supported that exercise training has positive effects in patients with schizophrenia: it enhances cognitive function and alleviates depressive psychiatric symptoms [45,46,47,48]. However, a study has revealed that high-intensity (85–95% HRR) aerobic exercise may reduce the participation rate of patients with severe schizophrenia [28]. Heggelund et al. recruited 25 younger patients with schizophrenia (mean age = 27 years) to participate in an eight-week intervention of high-intensity interval aerobic training (three sessions weekly, each session of 25 min), but six of their participants dropped out (attrition rate = 24%) [28]. Of note, we observed that none of the participants in the bench-step exercise group dropped out during the 12-week intervention, suggesting a relatively low-frequency training program could be effective to simultaneously alleviate cardiopulmonary fitness, attenuate depression levels, and improve the adhesion for training in patients with schizophrenia.

Our results indicate that our 12-week intervention of moderate-intensity (once per week) bench-step exercise training effectively improved overall depression level (reduced BDI-II score; Table 2). Our result is in line with previous studies that exercise training is capable of improving psychiatric symptoms of patients with schizophrenia [28,30,45,46,48]. For example, Vancampfort et al. (2012) discovered that lower 6MWD scores are correlated with negative and depressive psychiatric symptoms [30]. Moreover, another study employed an eight-week intervention of high-intensity interval aerobic training (three sessions weekly, each session of 25 min; the total number of sessions = 24, average weekly training duration = 72 min) alleviated the depressive symptoms of patients with schizophrenia [28].

However, in this study, the attention, measured by SMDT, did not exhibit overall improvements after bench-step exercise intervention. Possible reasons for this discrepancy include our relatively low sample size and relatively short intervention duration. Previous studies in which patients with schizophrenia achieved benefits on cognition employed higher training intensities and longer training durations (72–180 min weekly). For instance, Kimhy et al. adopted a 12-week intervention of moderate-intensity training (three sessions weekly, each session of 60 min; the total number of sessions = 36, average weekly training duration = 180 min) and reported significant improvements in aerobic fitness and MATRICS Consensus Cognitive Battery scores of patients with schizophrenia [48]. Therefore, future studies may include low-intensity exercise over longer intervention periods to verify whether this type of intervention can alleviate cognitive functions further.

SDMT total scores represent the participants’ selective attention capacity. However, in our exercise-training group, these scores did not exhibit significant differences compared with that of the control group. Stroth et al. (2009) reported that continuous aerobic exercise for 6 weeks (three sessions weekly, each session of 30 min) significantly enhanced the memory-related cognitive function performance in generally healthy adults [49]. Thus far, few intervention studies have evaluated the influence of endurance training on attention in patients with schizophrenia [50,51]. Malchow et al. (2015) employed three months of bundle intervention that included low-intensity cycling endurance training (lactic acid concentration of 1.5–2 mmol/L; three sessions weekly, each session of 30 min) and computer-assisted cognitive remediation (CACR) (two sessions weekly, each session of 30 min; initiated at the sixth week of training) [50]. Their results revealed that, between the sixth week and the final day of the three-month intervention, the cognitive function performance of patients with schizophrenia who received bundle intervention (i.e., endurance training and CACR) was enhanced significantly [50]. Despite the training intensity employed in this study is higher than that used by Malchow et al. [50], we did not observe a significant enhancement in cognitive function. This is possibly because Malchow et al. (2015) included additional CACR intervention, which might further enhance training diversity and complexity. Moreover, the training frequencies and total weekly training durations differed between our present study and that of Malchow et al. (2015). This indicates that the employed exercise volume to a total of 90 min/week could significantly enhance the neurocognitive function of patients with schizophrenia [46]. Therefore, a more diverse and greater training volume, as used by Malchow et al. (2015), can enhance the cognitive function of patients with schizophrenia further.

There are still some limitations to this pilot study. First, we allocated the participants into their treatment group based on their preference but not randomization, which might lead to potential selection biases and baseline differences in the psychological and pathological conditions of the included patients. Second, we measured their cardiopulmonary fitness using 6MWD instead of maximum oxygen uptake (VO_2_max), thus 6MWD might not provide a direct fitness assessment. Clinically, 6MWD was suggested as a reliable assessment tool for evaluating physical fitness in the population with schizophrenia [4]. However, future studies employing more rigorous and direct aerobic assessments, such as maximal oxygen uptake or ventilator/lactate threshold intensity, are warranted to better evaluate the improvements in aerobic endurance in population with schizophrenia. Third, because endurance training requires longer washout periods to achieve long-term effects, our exercise intervention cannot be investigated through blind testing or in crossover studies. A final limitation could be that the general control group was not included, but we have to note that the primary purpose of this study was to evaluate the benefit of bench-step training on the fitness and mental state of patients with schizophrenia. Further, because each study included patients who may be using different dosages of different antipsychotic agents, the psychological effects of drug treatment were excluded. We did not include a balance control to accommodate these effects.

## 5. Conclusions

This study demonstrated that the 12-week intervention of moderate-intensity bench-step exercise training (BSET; frequency: 1 session/week; each session of 30 min; step cadence: 96 beats/min) might effectively enhance the aerobic endurance and mood state of patients with chronic schizophrenia. However, their attention did not change after the BSET intervention. We, therefore, suggest that future studies are warranted to conduct long-term moderate-intensity exercise interventions and further verify the possible effects of such interventions on improving cognitive functions such as attention.

## Figures and Tables

**Table 1:** Basic population characteristic distribution of all participants.

Variable	Total Number *N* (%)	BSET Group *N* (%)	CTRL Group *N* (%)	*p*-Value
**Number**	28 (100%)	14 (50%)	14 (50%)	
**Height (cm)**	**-**	**168.1 ± 7.7**	**164.4 ± 7.9**	***0.111***
**Body weight (kg)**	**-**	**69.4 ± 10.9**	**67.1 ± 14.6**	***0.324***
**Gender**				***0.1***
Male	14 (50%)	9 (64.3%)	5 (35.7%)	
Female	14 (50%)	5 (35.7%)	9 (64.3%)	
**Age (yrs)**	**-**	**52.1 ± 11.0**	**50.1 ± 8.3**	***0.296***
21–30 yrs	1 (3.6%)	1 (7.1%)	0 (0%)	
31–40 yrs	2 (7.1%)	1 (7.1%)	1 (7.1%)	
41–50 yrs	9 (32.1%)	3 (21.4%)	6 (42.9%)	
51–60 yrs	10 (35.7%)	5 (35.7%)	5 (35.7%)	
61–65 yrs	6 (21.4%)	4 (28.6%)	2 (14.3%)	
**BMI (kg/m^2^)**	**-**	**24.7 ± 4.6**	**24.7 ± 3.8**	***0.489***
18 ≤ BMI < 24	12 (42.9%)	6 (42.9%)	6 (42.9%)	
24 ≤ BMI < 31	16 (57.1%)	8 (57.1%)	8 (57.1%)	
**SDMT (task scores)**	**-**	**30.7 ± 9.8**	**33.2 ± 10.7**	***0.*** ***252***
11–20 scores	3 (10.7%)	1 (7.1%)	2 (14.3%)	
21–30 scores	10 (35.7%)	6 (42.9%)	4 (28.6%)	
31–40 scores	10 (35.7%)	5 (35.7%)	5 (35.7%)	
41–50 scores	3 (10.7%)	1 (7.1%)	2 (14.3%)	
51–60 scores	2 (7.1%)	1 (7.1%)	1 (7.1%)	
**BDI-II (score)**	**-**	**11.3 ± 11.6**	**10.9 ± 9.5**	***0.*** ***459***
0–13 Minimal	17 (60.7%)	9 (64.3%)	8 (57.1%)	
14–19 Mild	3 (10.7%)	0 (0%)	3 (21.4%)	
20–28 Moderate	7 (25%)	4 (28.6%)	3 (21.4%)	
29–63 Severe	1 (3.6%)	1 (7.1%)	0 (0%)	
**6MWD (meters)**	**-**	**402.4 ± 78.9**	**418.1 ± 55.3**	***0.*** ***266***
<300 m	1 (3.6%)	1 (7.1%)	0 (0%)	
301–374.9 m	7 (25%)	3 (21.4%)	4 (28.6%)	
375–449.5 m	11 (39.3%)	5 (35.7%)	6 (42.9%)	
>450 m	9 (32.1%)	5 (35.7%)	4 (28.6%)	

BMI, body mass index; SDMT, Symbol Digit Modalities Test; BDI-II, Beck Depression Inventory-II; 6MWD, Six-Minute Walk Distance Test.

**Table 2:** Cardiopulmonary fitness, mood state, and attention.

Item	Group	PRE (Baseline)	POST (End)	*Time*	*Treatment*	*Interaction*	*within p*-Value	*Cohen’s d ES*
**6MWD (meters)**	*BSET*	402.4 ± 78.9	436.4 ± 81.5 *	*p < 0.01*	*p = 0.88*	*p = 0.15*	*p < 0.01*	*0.424*
	*CTRL*	418.1 ± 55.3	428.4 ± 71.8		(*n.s.*)	(*n.s.*)	*p = 0.18*	*0.160*
**BDI-II (scores)**	*BSET*	11.3 ± 11.6	8.8 ± 9.6 *	*p < 0.05*	*p = 0.85*	*p = 0.14*	*p = 0.03*	*0.235*
	*CTRL*	10.9 ± 9.5	10.6 ± 8.7		(*n.s.*)	(*n.s.*)	*p = 0.31*	*0.032*
**SDMT (tasks)**	*BSET*	30.7 ± 9.8	33.6 ± 11.7	*p = 0.07*	*p = 0.72*	*p = 0.27*	*N/A*	*N/A*
	*CTRL*	33.2 ± 10.7	33.9 ± 12.1	(*n.s.*)	(*n.s.*)	(*n.s.*)	*N/A*	*N/A*

* denotes the difference between PRE and POST within the group (*p* ≤ 0.05). ES, effect size; SDMT, Symbol Digit Modalities Test; BDI-II, Beck Depression Inventory-II; 6MWD, Six-Minute Walk Distance Test; *n.s.*, non-significance within group (*p* > 0.05).

## Data Availability

The data presented in this study are available upon request from the corresponding author.
